# Analysis of the situations and influencing factors of public anxiety in China: based on Baidu index data

**DOI:** 10.3389/fpubh.2024.1360119

**Published:** 2024-04-24

**Authors:** Tiantian Xie, Zetao Huang, Yue Tan, Tao Tan

**Affiliations:** ^1^Institute of New Rural Development, South China Agricultural University, Guangzhou, China; ^2^Centre de Recherche Sur Les Liens Sociaux (CERLIS), Université Paris Descartes, Paris, France; ^3^Institute of Biomass Engineering, South China Agricultural University, Guangzhou, China; ^4^School of Marxism, Chongqing Three Gorges Medical College, Chongqing, China

**Keywords:** public anxiety, Baidu index, spatial–temporal analysis, influencing factors, China

## Abstract

**Background:**

Anxiety disorders have emerged as one of the most prevalent mental health problems and health concerns. However, previous research has paid limited attention to measuring public anxiety from a broader perspective. Furthermore, while we know many factors that influence anxiety disorders, we still have an incomplete understanding of how these factors affect public anxiety. We aimed to quantify public anxiety from the perspective of Internet searches, and to analyze its spatiotemporal changing characteristics and influencing factors.

**Methods:**

This study collected Baidu Index from 2014 to 2022 in 31 provinces in mainland China to measure the degree of public anxiety based on the Baidu Index from 2014 to 2022. The spatial autocorrelation analysis method was used to study the changing trends and spatial distribution characteristics of public anxiety. The influencing factors of public anxiety were studied using spatial statistical modeling methods.

**Results:**

Empirical analysis shows that the level of public anxiety in my country has continued to rise in recent years, with significant spatial clustering characteristics, especially in the eastern and central-southern regions. In addition, we constructed ordinary least squares (OLS) and geographically weighted regression (GWR) spatial statistical models to examine the relationship between social, economic, and environmental factors and public anxiety levels. We found that the GWR model that considers spatial correlation and dependence is significantly better than the OLS model in terms of fitting accuracy. Factors such as the number of college graduates, Internet traffic, and urbanization rate are significantly positively correlated with the level of public anxiety.

**Conclusion:**

Our research results draw attention to public anxiety among policymakers, highlighting the necessity for a more extensive examination of anxiety issues, especially among university graduates, by the public and relevant authorities.

## 1 Introduction

Anxiety affects all people and has emerged as a pervasive collective psychological phenomenon within society, consistently retaining its position as one of the most prevalent and debilitating mental disorders worldwide. Some researchers have found that anxiety disorders have a significant impact on health, physical well-being, and overall economic productivity (1). Hunt characterizes anxiety as a consequence of precarious circumstances and a result of societal changes (2). In contrast, Sasaki defines anxiety as a communicative outcome resulting from evaluative concerns and impression formation during dichotomous social interactions. Additionally, it can be viewed as a prevailing societal mindset unique to specific historical periods (3).

During periods of significant societal change, anxiety often intensifies and spreads across diverse segments of the population. For example, students wrestle with anxiety related to their academic pursuits and future career prospects (4), while white-collar professionals face anxiety stemming from job pressures (5). Middle-aged individuals also grapple with anxiety, which may include concerns about their children’s education, older adult/adults parental care, and healthcare security, among other issues (6).

Since China initiated economic reforms and implemented the open-door policy in 1978, the nation has experienced rapid economic growth and profound socio-cultural changes (7). However, despite this rapid economic growth, the mental well-being and overall vitality of Chinese citizens have shown little improvement. The prevalence of mental disorders, especially anxiety disorders, has increased significantly, making anxiety a widespread social phenomenon (8).

The quantitative study of public anxiety extends prior research efforts empirically. Previous research has confirmed that social-economic factors, such as societal changes, social risk, social security, and technological progress, influence public anxiety (9, 10). However, these studies draw mainly on theoretical frameworks from sociology and social psychology based on real-world societal experiences and perceptions. Empirical research in this field still significantly lags.

Unlike individual anxiety, macro or public anxiety is an inherent aspect of social structures impacting diverse demographic groups. The significant impact of social and economic factors on societal-level anxiety is widely recognized (11, 12). Variations in the environment, including the Air Quality Index (AQI) among other factors, may also contribute to fluctuations in public anxiety levels (13–15). However, there has been relatively limited research on understanding how these factors interact comprehensively to shape public anxiety.

Previous research has often relied on subjective questionnaires to assess anxiety levels. However, these methods can be subject to bias due to individual psychological factors, cognitive processes, and societal expectations. The social stigma associated with mental health issues may cause some survey respondents to hide their true feelings when completing questionnaires.

In addition, according to the latest annual report of the China Internet Network Information Center (CNNIC) (16), the number of Internet users in China has grown steadily over the past decade, reaching 1.067 billion by the end of 2022. Online search has become an important means for most people to obtain information. The Baidu search engine has a dominant market share of over 70% in China, firmly establishing itself as the country’s primary search engine (17–19).

The Baidu Index (BDI) established based on Baidu search information can reflect the search needs and search awareness of most Chinese Internet users. There have been considerable studies utilizing BDI for public health research within China. For example, the BDI in Yunnan Province can be used to predict the incidence of chickenpox during the same period, indicating that the BDI is a useful tool for monitoring chickenpox epidemics and complementing traditional surveillance systems (18). Some researchers used BDI data to reveal the spatiotemporal distribution of migraine in China (20). The tuberculosis prediction model in Jiangsu Province, China, based on Baidu Index data, can predict the next wave of tuberculosis epidemic trends and intensity 2 months in advance (19). The Baidu Index can also be used to analyze the distribution of asthma prevalence in China, indicating that the overall prevalence is on an upward trend (21).

The introduction of the BDI provides a valuable opportunity to quantitatively measure public anxiety by analyzing Internet search behavior, a form of large-scale data commonly referred to as big data (22). The main goal of this research is to operationalize the measurement of public anxiety using Internet search behavior data and to quantitatively investigate the factors that influence public anxiety, including macro-level indicators. The Internet allows individuals to protect their privacy, leading to more authentic behavior. This approach has the potential to reveal information that is difficult to capture through traditional academic surveys, providing a more accurate measurement of anxiety levels (23–25). In addition, conducting research at the provincial administrative level while investigating macro-level mechanisms that influence public anxiety contributes to improved decision-making around social governance. Considering these considerations, this study aims to thoroughly measure the extent, spatiotemporal patterns, and evolutionary path of public anxiety in China. In addition, it aims to investigate the related influencing factors and mechanisms.

## 2 Materials and methods

### 2.1 Study area

The study encompasses the 31 provincial-level administrative regions situated in mainland China, including 22 provinces, 4 centrally administered municipalities, and 5 autonomous regions. However, due to data constraints, Hong Kong, Macau, and Taiwan are not within the scope of this research.

### 2.2 Data sources

#### 2.2.1 Baidu index

The BDI serves as the official repository and data sharing platform associated with the Baidu search engine, providing insight into the behavioral patterns of Baidu users. It provides comprehensive insights into the keywords that appear in Internet search queries. The Baidu Index uses search volume as its base data, calculating the weighted sum of search frequencies for specific keywords on the Baidu search engine. This measure indicates the extent to which Internet users access and search for information on a particular topic within a given period. It is important to recognize that while there are challenges in directly measuring anxiety, keyword searches reflect subjective choices driven by concerns about specific issues. Users may have personally experienced these issues or may be motivated by the experiences of family members and friends who are dealing with psychological challenges. Consequently, these circumstances prompt individuals to initiate searches for these keywords on Baidu search engine, either to deepen their understanding or to seek psychological support.

By comparing the Baidu Index of keywords such as “worry” and “anxiety,” we found that “anxiety” has become the most used typical keyword by Chinese netizens when conducting anxiety-related searches. Given the essential considerations of scientific validity, conciseness, and operational feasibility, we have carefully chosen “anxiety” as the primary search term in the BDI framework. The rationale for this choice is multifaceted. The chosen keyword should ideally represent typical user search behavior, demonstrating strong representativeness, robust search volume, and comprehensive coverage of search content. However, using all relevant terms in this way would result in an unmanageable amount of data and complex data relationships, making comprehensive analysis difficult. When Internet users seek information about the various aspects of this condition, its causes, and potential remedies, “anxiety” is consistently the first term they enter the Baidu search engine. Moreover, the calculation method of the Baidu Index will include any input sentences containing this keyword into statistics. Therefore, while we acknowledge the potential limitations of using “anxiety” exclusively as the sole keyword, it is the most prudent approach currently. Furthermore, based on this explanation, we assert that the data obtained through the BDI in this manner can effectively reflect the current level of public anxiety within the Chinese population.

The original data of BDI can be furnished based on specific temporal intervals and geographic regions. This data enables the examination of contemporary trends in search volume and spatiotemporal distribution characteristics among Chinese Internet users, specifically pertaining to the theme of “anxiety.” However, the alteration in the measurement algorithm in 2013 necessitates caution, as data gathered before and after this pivotal date are not directly comparable. Furthermore, during the period spanning 2011 to 2013, the distinctions in search index values among various provinces were exceedingly negligible, posing a substantial challenge for statistical analysis. Consequently, to comprehensively portray the magnitude of public anxiety and facilitate spatial econometric modeling, we have opted to employ the provincial-level annual average Baidu index (ABDI) for 2014 to 2022 as the dependent variable.

#### 2.2.2 Socioeconomic and environmental data

Public anxiety levels are influenced by the multifaceted interaction of economic, environmental and contextual factors. The rapid economic development and urbanization have improved people’s living standards and raised people’s expectations for future life. People strive to constantly meet their own expectations and therefore experience more mental stress. The rapid development of Internet technology during the period of social transformation has accelerated the spread of social anxiety. With technological advancement, population growth, and people’s ever-improving pursuit of life, human intervention in the natural world has become more and more serious, resulting in climate change. This study selected nine relevant variables as potential explanatory factors based on other literature practices and data availability (9, 12, 26–28).

The rapid economic development and urbanization have raised people’s expectations for future life. People strive to constantly meet their own expectations and therefore experience more mental stress. GDP *per capita* (GDPP) is the level of economic development that has a substantial impact on living standards and access to Internet resources.

Urbanization rate (UR) may encourage the development of infrastructure, foster cultural environments, and impact spiritual well-being, which could potentially affect public anxiety. We use the urbanization rate to measure urbanization levels.

Population density (PD) impacts the rate and extent of regional communication. Areas with dense populations experience a quicker and wider spread of public anxiety among residents.

Unemployment rate (UER), which reflects the percentage of jobless individuals in the overall population, indicates heightened societal unemployment. This situation can lead to increased stress and anxiety about the future.

Additionally, the number of college graduates (NCG) in a region can be a factor in determining the economic and social well-being of an area. Studies reveal that a substantial proportion of graduates undergo anxiety symptoms when making the transition from academia to the workforce. Additionally, since university students tend to use the internet more frequently, they are more inclined to express their anxiety online. Thus, we have included the annual count of college graduates as an explanatory factor to examine its possible correlation with public anxiety.

Regarding internet data traffic (IDT), previous studies often employed internet penetration rates to depict societal informatization levels, while we opted to use total internet data traffic as a more intuitive metric. Thus, we measure the extent of internet development using the annual total internet data traffic. The internet has greatly increased accessibility to information and interpersonal communication, reducing the negative impacts of geographic distance. It has also improved individuals’ ability to address their own needs and express emotions online, making it a key component in the study of public anxiety trends.

We also gathered precipitation (PT) and annual average temperature (AT) data for the same period and provinces to investigate the possible effects of temperature variations on public anxiety.

In accordance with the current standards in China, we use the AQI to provide a more comprehensive evaluation of air pollution levels. Our data collection method for the AQI is identical to that used for precipitation and annual average temperature.

To acquire provincial-level data, we extract resources from the website of the China National Bureau of Statistics (29) along with data from the respective provincial statistical bureaus’ statistical yearbooks (30). We access temperature, precipitation, and AQI data from the China Air Quality Online Monitoring and Analysis Platform (31).

### 2.3 Spatial analysis

#### 2.3.1 Geographic concentration index and disequilibrium index

In this study, we utilize the Geographic Concentration Index (GCI) and Geographic Disequilibrium Index (GDI) to analyze the spatial distribution patterns of public anxiety in China. The GCI functions as a tool to measure the concentration level of a given phenomenon across a variety of geographic regions (32, 33) and in this case, is applied to gauge the degree of concentration of public anxiety throughout China. Equation (1) provides the calculation formula for the GCI.

(1) $$G=100\times \sqrt{\overset{n}{\underset{i=1}{\sum }}{\left(\frac{X_{i}}{T}\right)}^{2}}$$

In equation (1), $X_{i}$ is the total attention in the region, *T* is the total attention across all regions, *n* is the number of regions considered, and *G* is the GCI. It’s important to note that a higher GCI value indicates a greater concentration of attention, indicating an uneven distribution. Conversely, a lower GCI value indicates a more dispersed pattern of attention, suggesting a more balanced distribution.

The GDI serves as a tool to measure the degree of geographic disparity in the distribution of the research subject across different levels or regions (34). The mathematical formula is shown in equation (2).

(2) $$S\;=\;\frac{\sum_{i=1}^{n}\;Y_{i}-50\;\left(n+1\right)}{100\times n-50\;\left(n+1\right)}$$

In equation (2), *S* represents the GDI of BDI, a value ranging from 0 to 1. In addition, *i* represents the total number of provinces, and $Y_{i}$ represents the cumulative percentage of BDI in the region after being sorted in descending order.

#### 2.3.2 Spatial autocorrelation test

In this study, we employ spatial autocorrelation analysis to explore the similarity and spatial correlation patterns of BDI data associated with public anxiety in nearby regions. To gauge the spatial autocorrelation and heterogeneity in public attention across neighboring areas, we utilize the Global Moran’s I index (35). Equation (3) presents the calculation formula.

(3) $$I=\frac{\sum_{i=1}^{n}\sum_{j=1}^{n}w_{ij}\left(x_{i}-\bar{x}\right)\left(x_{j}-\bar{x}\right)}{\sigma^{2}\sum_{i=1}^{n}\sum_{j=1}^{n}w_{ij}}$$

In equation (3), *n* represents the number of provincial-level administrative regions, $x_{i}$ and $x_{j}$ represent the BDI for two different regions, respectively. The variable $\bar{x}$ represents the average BDI across all regions, $\sigma^{2}$ represents the variance, and $w_{ij}$ represents the spatial weight matrix.

The spatial weight matrix is a representation of the spatial structure of data. It is a quantification of the spatial relationships that exist between features in a data set. In relevant research (36–38), the inverse distance method or inverse distance squared method is usually used. The inverse distance method is best suited for continuous data, or for modeling objects where the closer two features are in space, the more likely they are to interact or influence each other. The inverse distance squared method is the same as the inverse distance method, except that the slope is steeper, so the influence drops off faster, and only the nearest neighbors of the target feature have a significant impact on the calculation of that feature. In order to confirm the robustness of the constructed spatial autocorrelation model. We calculated using the inverse distance and inverse distance squared method, respectively.

Equation (4) provides the *Z*-test statistic, which is used to determine the significance of the Global Moran’s I index. If *|Z|* exceeds 1.65, it indicates a significance level below 0.10. Similarly, if *|Z|* exceeds 1.96, it corresponds to a significance level below 0.05. Additionally, if *|Z|* exceeds 2.58, it implies a significance level below 0.01. These thresholds are commonly used in statistical hypothesis testing to determine the significance of research results.

(4) $$Z=\frac{I-E\left(I\right)}{\sqrt{Var\left(I\right)}}$$

Although the Global Moran’s I index offers a national-level overview of the general spatial clustering of the BDI, it does not provide the ability to delineate the specific distribution of clusters. Therefore, we employ the Local Moran’s I index to gain insights into the spatial agglomeration and differentiation characteristics (35). Equation (5) furnishes the formula for calculating the Local Moran’s I index.

(5) $$I_{i}=z_{i}\overset{n}{\underset{i\neq j}{\sum }}w_{ij}z_{j}$$

In equation (5), $I_{i}$ is the Local Moran’s I index for the regions, $z_{i}$ and $z_{j}$ denote the standardized values of the BDI for the regions, respectively. $w_{ij}$ is the spatial weight matrix.

The Local Moran’s I index for a province can be classified into one of four clustering types: High-High, Low-Low, High-Low, or Low-High cluster, depending on its positive or negative value. These classifications help identify whether a province exhibits spatial clustering tendencies with its neighboring regions regarding BDI values.

### 2.4 Spatial econometric models

To assess the effect of social, economic, and other factors on public anxiety, this study performs a comparative analysis of two econometric models. The OLS algorithm (39) is employed to evaluate the impact of nine independent variables on public anxiety. The resulting model is represented by equation (6).

(6) $$y=\beta_{0}+\beta_{i}x_{i}+\varepsilon$$

In equation (6), *y* is the dependent variable representing the BDI for anxiety. The parameters $\beta_{i}$ denote the coefficients for the independent variables $x_{i}$ , all of which are defined as natural logarithms. Furthermore, $\beta_{0}$ refers to the intercept term, while *ε* represents the error term.

Tobler’s First Law of Geography emphasizes spatial correlation in spatial data, where proximity strengthens spatial relationships. Thus, spatial data’s inherent correlation can introduce spatial heterogeneity into regression models, unlike conventional cross-sectional data. GWR models offer a persuasive framework for spatial research. Unlike OLS models, GWR models effectively analyze the spatial non-stationarity present in spatial data. The GWR model has demonstrated promising applications across various disciplines (37, 38, 40). However, OLS models used in similar studies have neglected spatial correlations among variables, which can lead to estimation bias (41). In order to conduct a more thorough analysis of the factors influencing public anxiety, we use the GWR model, addressing this concern comprehensively. Equation (7) summarizes the GWR model concisely.

(7) $$Y_{i}=\beta_{0}\left(u_{i},v_{i}\right)+\overset{p}{\underset{j=1}{\sum }}\beta_{j}\left(u_{i},v_{i}\right)X_{ij}+\varepsilon_{i}$$

In equation (7), $Y_{i}$ is the dependent variable, $\beta_{0}$ is the intercept, $u_{i}$ and $v_{i}$ are the longitude and latitude respectively, $\beta_{j}\left(u_{i},v_{i}\right)$ denotes the spatial geographical location function, $X_{ij}$ is the value of the *j*-th explanatory variable at the *i*-th sample point, and $\varepsilon_{i}$ signifies the random error.

Multicollinearity is characterized by elevated correlations among explanatory variables, potentially resulting in ineffective parameter estimates, inconclusive significance tests, and diminished predictive accuracy within models. Due to its common occurrence in modeling, it’s essential to assess multicollinearity among explanatory variables and, if necessary, eliminate variables that contribute to multicollinearity before modeling (42, 43). The formula for calculating the Variance Inflation Factor (VIF) for an explanatory variable is (equation 8).

(8) $$VIF=\frac{1}{1-{R_{i}}^{2}}$$

In equation (8), $R_{i}$ is the negative correlation coefficient of an explanatory variable when regressed against the other explanatory variables. A high VIF value indicates an increased likelihood of multicollinearity among the explanatory variables. Typically, when the VIF exceeds 10, it signals a noteworthy multicollinearity issue in the regression model. According to widely accepted criteria for detecting multicollinearity in many studies (42, 43), a VIF below 10 is considered acceptable. Hence, the modeling process can be executed when there is no significant multicollinearity among the explanatory variables.

## 3 Results

### 3.1 Spatial–temporal variation of public anxiety

#### 3.1.1 Temporal evolution characteristics

**Figure 1:**
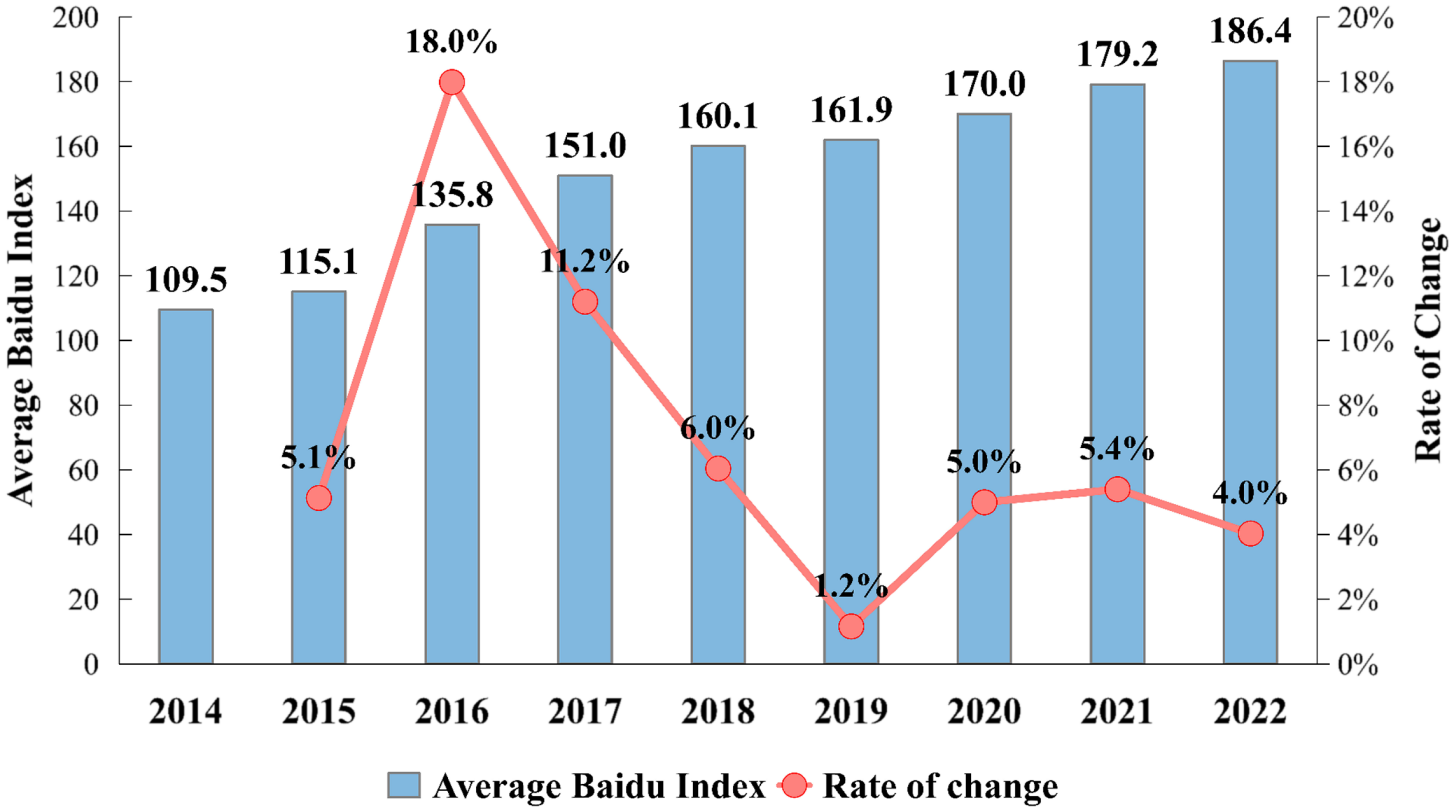
China’s national-level ABDI during 2014–2022.

Throughout the entire study period, the ABDI consistently showed year-on-year increases. Figure 1 displays the ABDI for “Anxiety” and its growth rate from 2014 to 2022, with detailed results available in Supplementary material. In 2016, it experienced a significant 18% growth rate, nearly reaching 1.5 times its 2014 level by 2017. This surge can be attributed to the widespread expansion of the internet in China during that time, leading to an overall increase in public internet search activity. From 2018 to 2022, the ABDI exhibited a fluctuating yet upward trajectory. In 2019, the growth rate was a modest 1.2%, and by 2022, it had reached 4.0%. Overall, the BDI consistently remained at a high level, indicating a strong upward trend in public anxiety.

**Figure 2:**
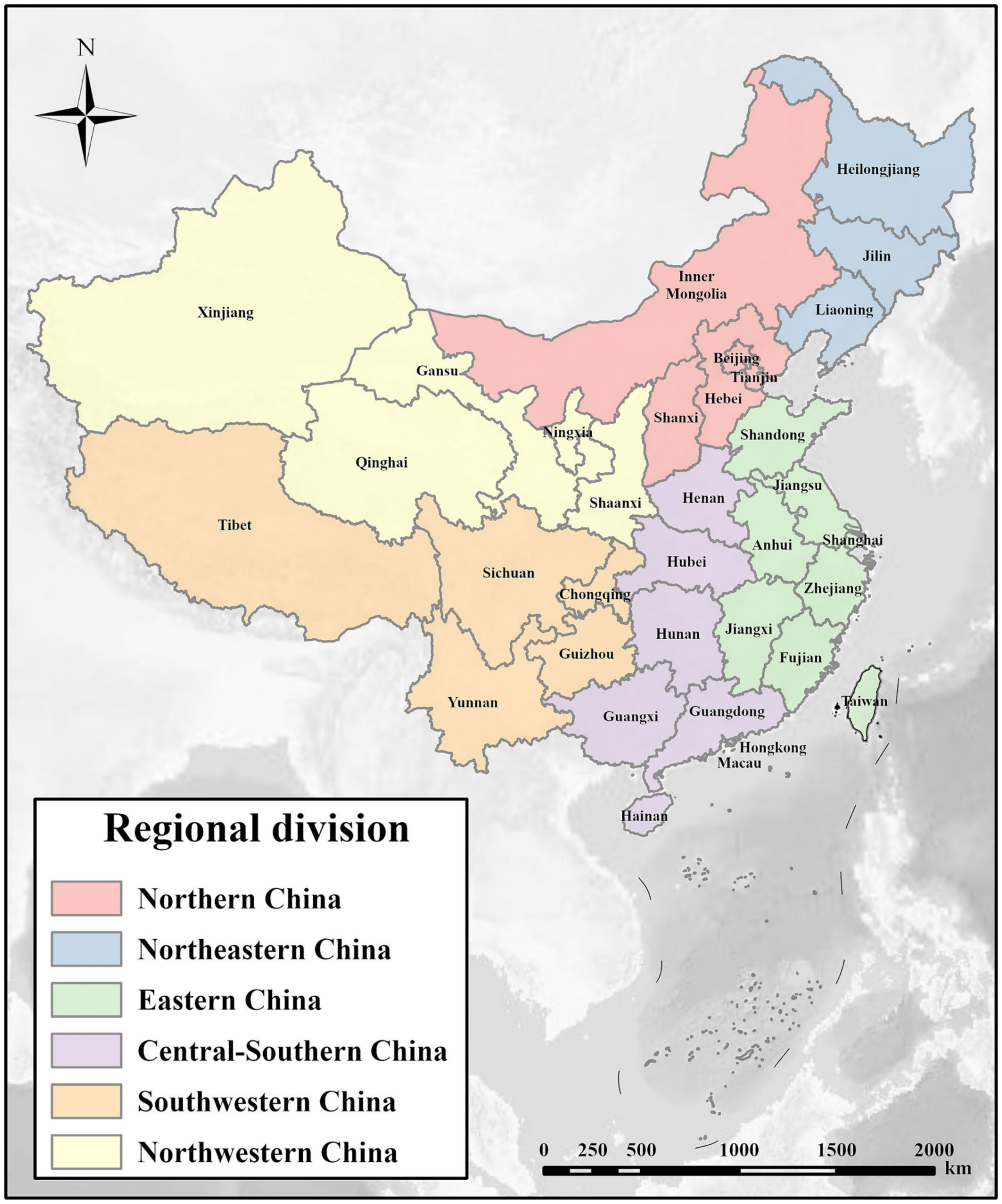
Map of China’s regional division.

Figure 2 illustrates the six primary regions in China as defined by the National Bureau of Statistics. Notably, considerable differences in ABDI are observable across these regions. Between 2014 and 2022, East China (which experienced a rise in ABDI from 139 to 229) and Central South (135 to 225) garnered more public attention, while Northwest (65 to 107) and Southwest (75 to 134) saw relatively lower levels of attention. Among the six regions in the study area, the growth rates of ABDI consistently exhibit a trend of initial increase followed by subsequent decrease in alignment with the national pattern. The growth rate began to decline in 2016, after a period of rapid expansion from 2015 to 2017.

The progression of the ABDI across the 31 provincial-level administrative regions is depicted in the Supplementary material. Notably, the provinces with the highest public attention include Guangdong (ABDI of 456), Jiangsu (293), and Shandong (291). In the early phase (2014–2017), Tibet (with an average annual growth rate of 74.4%), Qinghai (52.9%), and Ningxia (30.3%) experienced rapid ABDI growth. In the following period (2018–2022), Tibet (18.3%) and Guangdong (7.7%) maintained high ABDI growth. Additionally, in 2019, the Northern China was the only region in the country to undergo negative growth, with an annual growth rate of −0.7%. Specifically, within the provinces of the North China region, excluding Shaanxi, Tianjin (with a − 2.8% annual growth rate), Beijing (−2.0%), Hebei (−0.7%), and Inner Mongolia (−0.6%) all experienced negative growth during that year. In 2019, provinces outside the North China region, including Shanghai (−2.3%), Jiangxi (−1.5%), Guizhou (−0.9%), and Guizhou (−0.4%), also reported negative growth.

#### 3.1.2 Spatial distribution characteristics

**Figure 3:**
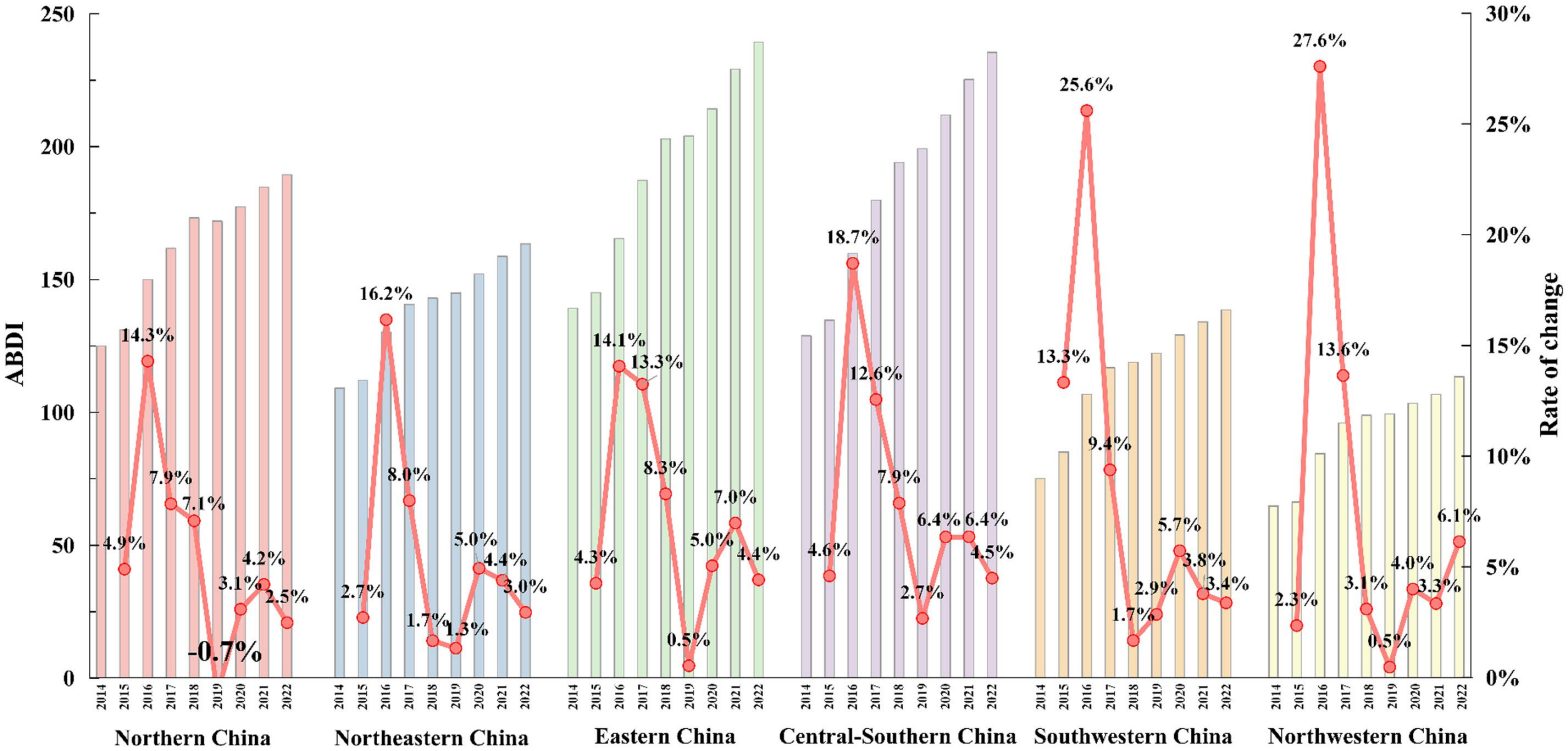
China’s regional-level yearly average BDI during 2014–2022.

**Figure 4:**
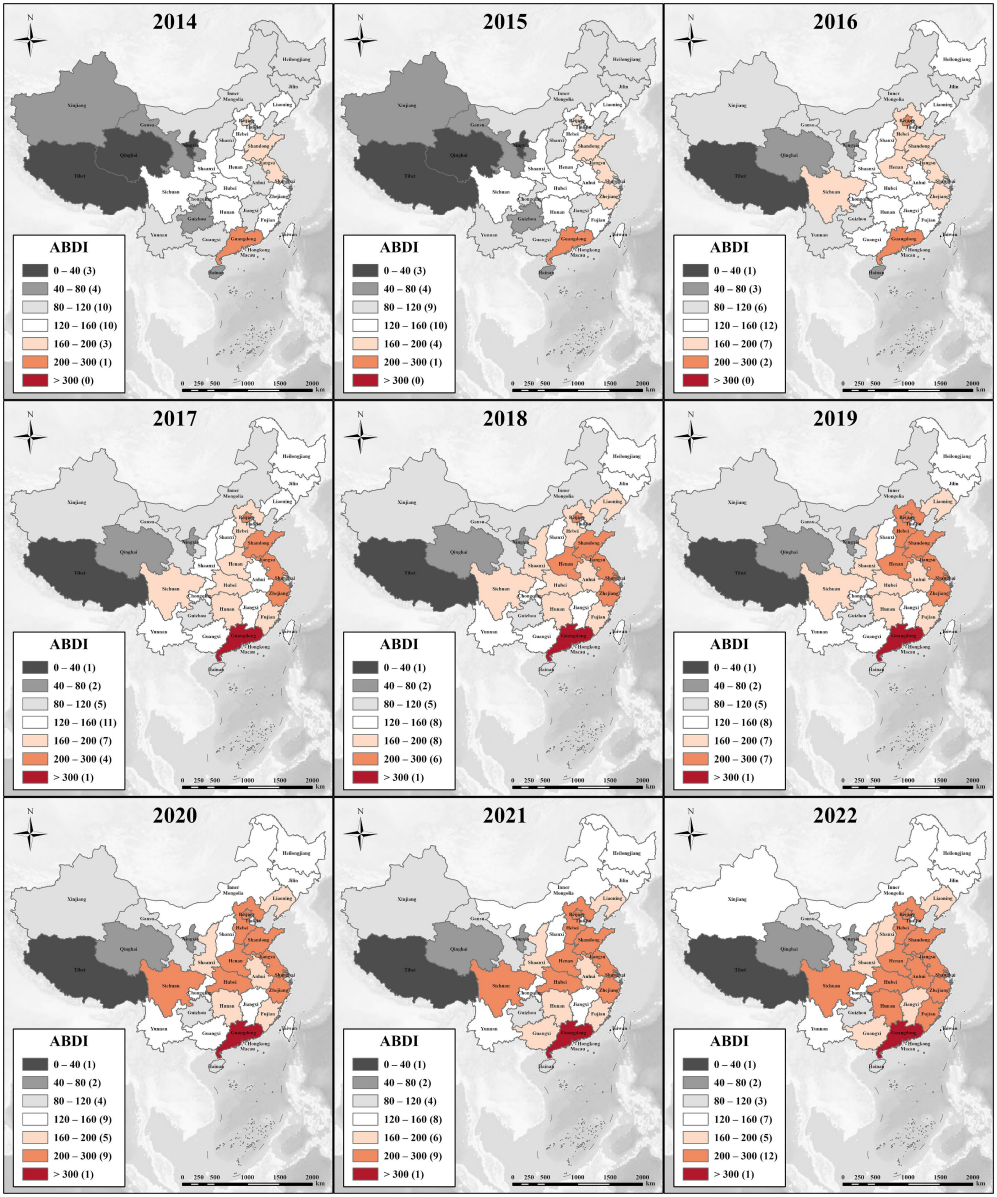
Spatial distribution of ABDI during 2014–2022.

Figure 3 shows the provincial variations of ABDI in China between 2014 and 2022. There is a recognizable spatial pattern emerging in the distribution of ABDI across China’s provinces, with the following order: Eastern > Central-Southern > Northern > Southwestern > Northeastern > Northwestern China. To provide a visual aid in clarifying this evolving pattern, Figure 4 presents the spatial distribution of ABDI from 2014 to 2022. Over time, the number of provinces with ABDI values of 160 or lower has decreased from 27 in 2014 to 13 in 2022, indicating a considerable reduction in regions with low attention levels. On the other hand, the quantity of regions with ABDI values exceeding 200 has risen from 1 in 2014 to 13 in 2022, signifying a significant increase in the number of regions with high attention levels. The BDI levels have substantially increased in Eastern China, Central-Southern, and Northern China. However, in the Northwestern and Southwestern regions of China, including Tibet, Qinghai, Ningxia, and Gansu, ABDI has remained relatively low.

**Figure 5:**
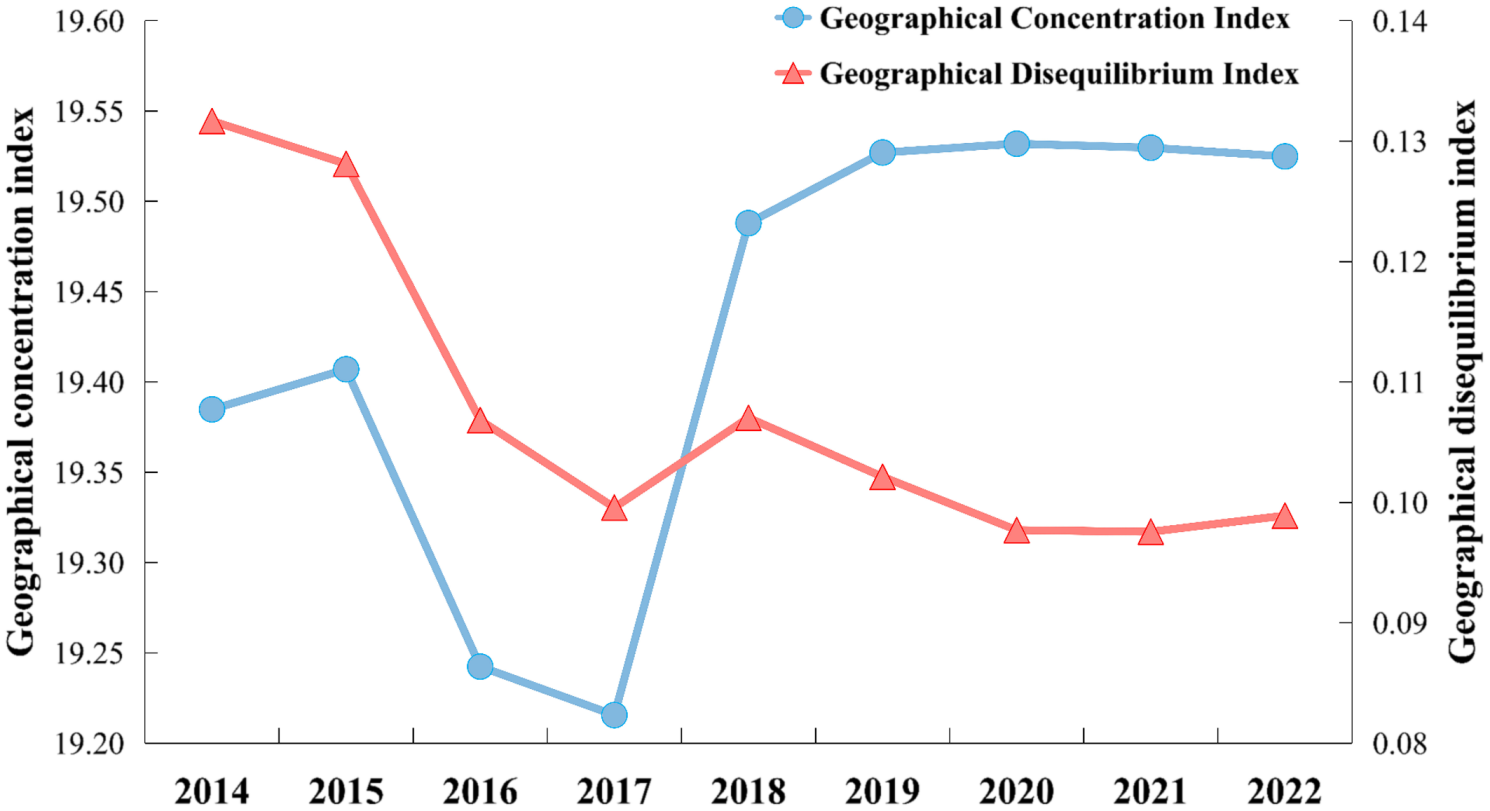
GCI and GDI of ABDI during 2014–2022.

Figure 5 shows the calculated results for the GCI and GDI from 2014 to 2022. Assuming an equal distribution of ABDI across the 31 regions in mainland China, an expected GCI result is 17.961. However, the actual calculations have consistently exceeded this value, ranging from 19.385 to 19.525, indicating a discernible upward trend. The results suggest a gradual increase in the concentration of the ABDI at the provincial level. In contrast, the GDI decreased from 0.132 to 0.099, indicating a significant and steadily increasing polarization in the distribution of ABDI.

In 2016 and 2017, the value of GCI dropped significantly. This is because these two years were the period when Internet penetration grew fastest. The ABDI growth rate of inland provinces is significantly faster than that of other provinces. This reduces the numerical differences between provinces, and the distribution concentration represented by GCI decreases accordingly. Subsequently, as the differences in development rates between regions narrowed, ABDI also showed more obvious regional concentration characteristics.

In the early stages, due to the gradual popularization of the Internet in economically underdeveloped areas, people had more opportunities to express their needs on the Internet. Therefore, the rapid growth of ABDI was detected early in the inland areas. In recent years, anxiety-related search behaviors have been mainly distributed in coastal provinces. This may indicate that the distribution of social anxiety levels is closely related to regional economic development and population density. However, this is only a result speculated based on changes in ABDI’s spatiotemporal distribution. Spatial statistical analysis methods need to be applied to test this speculation.

#### 3.1.3 Spatial autocorrelation analysis and spatiotemporal cluster analysis

Expanding on previous calculations, the spatiotemporal pattern of ABDI indicates spatial clustering, which suggests a spatial correlation between ABDI in one province and its neighboring provinces. To further explore these clustering patterns, we performed spatial autocorrelation and spatiotemporal clustering analyses. Throughout the research period, Table 1 presents the global spatial autocorrelation results of ABDI.

**Table 1:** Global Moran’s I index of BDI for “Anxiety” during 2014–2021.

Year	The inverse distance method			The inverse distance squared method		
	Global Moran’s index	*z*-score	*p*-value	Global Moran’s index	*z*-score	*p*-value
2014	0.3610	4.4974	<0.0001	0.3851	3.9629	<0.0001
2015	0.3357	4.2233	<0.0001	0.3622	3.7583	0.0002
2016	0.2627	3.4110	0.0006	0.2843	3.0394	0.0024
2017	0.2733	3.5480	0.0004	0.2953	3.1570	0.0016
2018	0.2479	3.2749	0.0011	0.2695	2.9278	0.0034
2019	0.2234	3.0153	0.0026	0.2432	2.6964	0.0071
2020	0.2134	2.9153	0.0036	0.2297	2.5812	0.0098
2021	0.2146	2.9318	0.0034	0.2306	2.5912	0.0096
2022	0.2135	2.9208	0.0035	0.2315	2.6018	0.0093

The inverse distance method and inverse distance squared method both show the same calculation results. However, the z-score value of the inverse distance method is better, showing that it has better statistical significance. Therefore, we use the results calculated by this method as the basis for subsequent analysis.

Significantly, the Global Moran’s I index consistently maintains a positive value exceeding 0.2, with z-values exceeding 2.58, indicating highly significant and stable spatial autocorrelation demonstrated by ABDI, meeting the 1% significance threshold. Therefore, the results suggest a strong positive correlation between ABDI values in provinces and regions and their neighboring counterparts.

**Figure 6:**
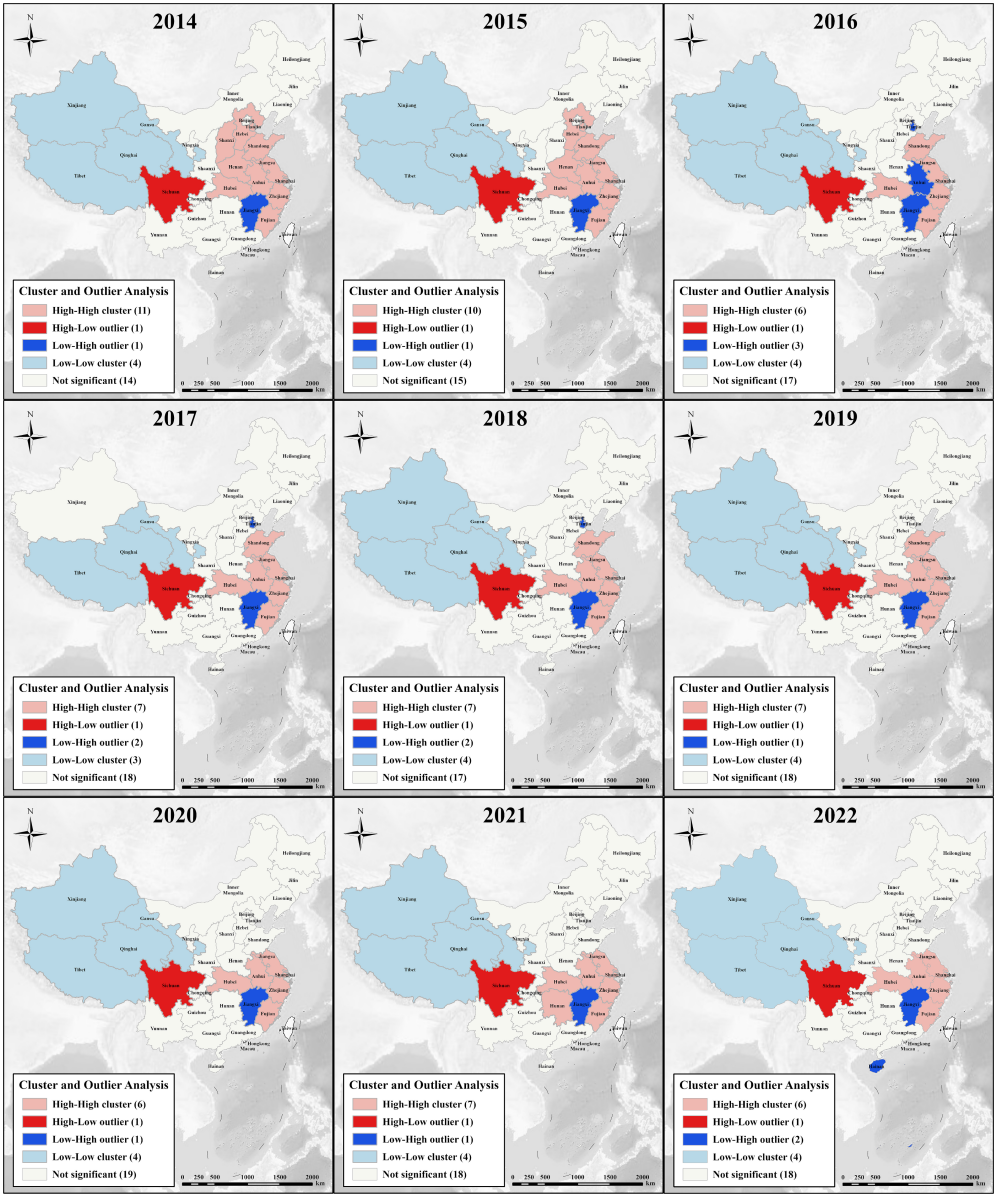
Cluster and outlier analysis of BDI for “Anxiety” during 2014–2022.

Moreover, we conducted a precise local spatial autocorrelation analysis to augment our comprehension of the spatial distribution characteristics of ABDI. The outcomes of the spatial autocorrelation and clustering analysis corroborate the noteworthy spatial clustering of public anxiety in China. These spatial clustering patterns are visually depicted in Figure 6. ABDI manifests four distinct clustering patterns in its regional distribution:

Low-Low Cluster: Situated in northwestern China, this cluster includes Xinjiang, Qinghai, Tibet, and Gansu (excluding Xinjiang in 2017).

High-High Cluster: This cluster underwent notable changes in 2017 and 2019, with Hebei, Shanxi, and Henan no longer included. The cluster’s center shifted southward, signifying an elevation in the BDI level in the East China region. As of 2022, the center of the High-High cluster has relocated to the southeastern part of China, encompassing major provinces in the East China and Central South regions.

Low-High Cluster: Over the study period, this cluster remained stable, predominantly comprising Jiangxi, with significantly lower ABDI than the surrounding regions.

High-Low Cluster: In 2017, Tianjin was categorized as a Low-High cluster, while Sichuan was designated as a High-Low cluster, deviating from the typical patterns.

### 3.2 Correlation analysis of influencing factors

#### 3.2.1 Selection of influencing factors

Consistent with established reference indicators and data availability in similar studies, our research incorporates nine pertinent variables as explanatory factors. We conduct a quantitative examination of these variables to elucidate their impact on ABDI and underlying mechanisms.

Table 2 displays the units, abbreviations, and definitions of the selected explanatory variables. The Supplementary material provide detailed variable statistics, including their respective Variance Inflation Factor (VIF) values utilized for evaluating multicollinearity. It is worth noting that all variables display VIF values below 10, confirming the adequacy of our model results.

**Table 2:** Definitions and descriptions of variables.

Variables	Labels	Units	Definitions
GDP *per capita*	GDPP	CNY	The gross domestic product divided by the population
Urbanization rate	UR	%	The proportion of urban population to total population
Population density	PD	Person/km2	The amount of population per unit area
Unemployment rate	UER	%	Proportion of the unemployed in the year
Number of college graduate	NCG	10,000 peoples	Number of college graduates in the year
Internet data traffic	IDT	10,000 Gigabytes	Total Internet traffic in the year
Precipitation	PT	Millimeter	Total precipitation of the year
Average temperature	AT	°C	Average temperature of the year
Air quality index	AQI	Value (0–500)	Average air quality index of the year

#### 3.2.2 Comparison of model goodness-of-fit

The differences in scale and units among the variables can affect the creation of regression models. Thus, we utilized the natural logarithm transformation for all variables before executing the regression analysis because it is a frequently used econometric approach. The outcomes of both the OLS and GWR models are summarized in the Supplementary material.

**Figure 7:**
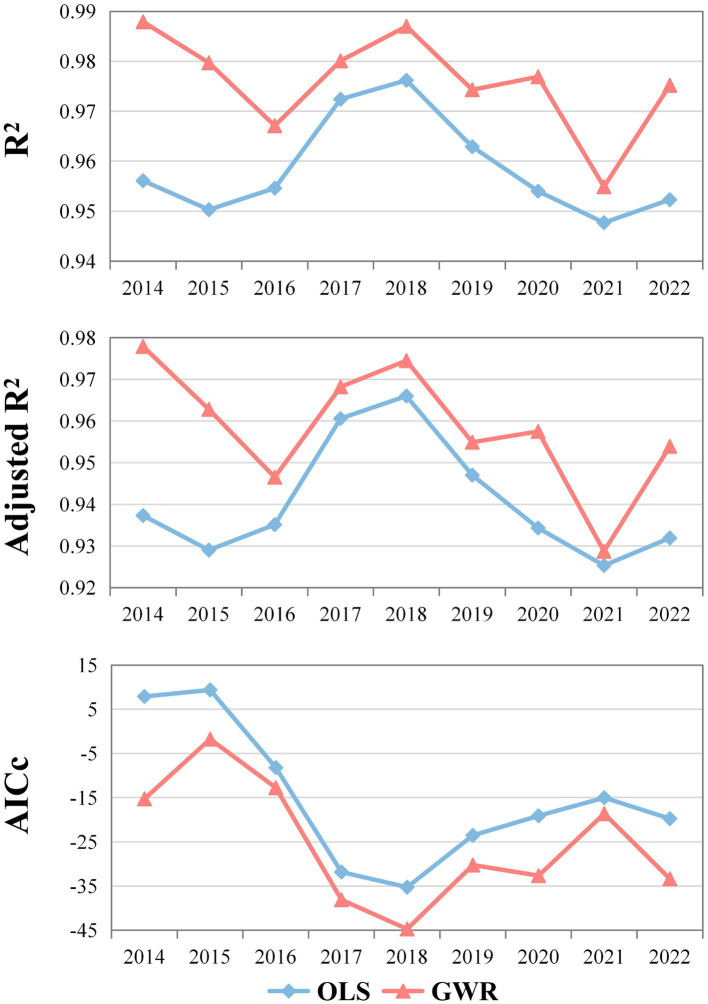
OLS and GWR model fitting results.

The OLS model had *R*-squared (*R*^2^) values of 0.9561 in 2014, 0.9762 in 2018, and 0.9523 in 2022. These values indicate that the OLS model has relatively strong explanatory power. However, the GWR model produced higher *R*^2^ values: 0.9879 in 2014, 0.9870 in 2018, and 0.9752 in 2022 (Figure 7). These results show that the GWR model, which accounts for spatial correlation and dependency effects, significantly outperforms the traditional OLS model. It’s worth noting that there are clear differences in the significance of the explanatory variables between the OLS and GWR models. For example, in 2021 and 2022, the OLS model results showed that only IDT passed the 1% significance level test, while the GWR model revealed that NCG and UR are also significant contributing variables. This disparity suggests that the OLS model may overlook the impact of certain variables.

Furthermore, when considering model fit indices, including *R*^2^, Adjusted *R*^2^, and AICc, for all years, the GWR model consistently outperforms the OLS model. It indicates that GWR provides a superior explanation of the relationships between variables. Consequently, our primary focus is on the results obtained from the GWR model.

#### 3.2.3 Empirical results and interpretations

In summary, during the research period spanning from 2014 to 2022, significant fluctuations were observed in both the coefficients and the significance levels of individual variables. Furthermore, there have been temporal shifts in the impact of explanatory variables on ABDI, and these variations will be elucidated in subsequent sections.

The analysis of the GDPP produced inconclusive results since neither the OLS nor GWR models showed consistently positive or negative coefficients. Therefore, it is not possible to establish a clear link between economic growth and public anxiety levels at the provincial level. Consequently, the initial hypotheses that suggested economic development and resident income gaps as the primary factors governing public anxiety lack support.

The PD coefficient ranged from 0.0026 to 0.1185 with little year-to-year variability. However, it was not statistically significant in any model year, suggesting that higher population density does not lead to increased ABDI. This result contradicts the previous assumption that population density plays a crucial role in online data acquisition and transmission.

The coefficient for UER showed a negative correlation with ABDI, ranging from −0.0832 to −0.2914. It is worth noting that statistical significance was found for UER for the years between 2017 and 2019, with particularly significant results in 2018 when the coefficient value exceeded the 1% significance level threshold at −0.2914. Therefore, a 1% increase in unemployment is linked to a decrease of 0.29% in ABDI. This unexpected discovery challenges the accepted notion that an increase in unemployment results in increased public unease.

In China, a distinct and strong positive link between NCG and ABDI is evident, with coefficients ranging from 0.1917 to 0.5888 over the duration of the study. These coefficients consistently display high levels of statistical significance, emphasizing NCG’s essential role as a primary determinant of ABDI amid the selected explanatory variables. Although attempts were made to replace the current count of university students with NCG, the results showed that NCG presented a more robust explanatory framework.

The influence of IDT paralleled that of NCG, with coefficients spanning from 0.0615 to 0.3209 across the entire research period. In contrast to NCG, IDT lacked statistical significance in 2014–2015 but notably gained substantial significance in 2016, maintaining this significance through 2022. It implies that the association between internet development and ABDI has grown stronger over time. Considering that the BDI is fundamentally rooted in publicly accessible internet search volumes, this observed relationship is consistent with our initial hypothesis.

PT coefficients ranged from −0.1395 to 0.0554, and neither model displayed a statistically significant correlation with ABDI. Likewise, AT had coefficients ranging from −0.1079 to 0.2249, but no significant correlation with ABDI was observed in either model. These findings suggest that climatic and temperature disparities among provinces do not lead to fluctuations in public anxiety, thereby contradicting our initial speculation.

UR had a positive influence on ABDI, and all GWR model outcomes showed statistical significance. From 2014 to 2022, the coefficients varied between 0.4274 and 1.5274, with a concurrent decrease in t-values from 1.9126 to 1.7053. It signifies a diminishing influence of UR on the strength and significance of ABDI. This phenomenon may be attributed to the rapid urbanization observed in China throughout the research period, which led to a reduction in disparities between provinces. China’s implementation of targeted poverty alleviation and economic development initiatives led to increasing similarities in the lifestyles of rural and urban residents, thereby resulting in similar mechanisms and levels of public anxiety for both groups.

The results for AQI mirrored those of GDPP, exhibiting inconclusive coefficients and lacking significance across all years. This inconsistency with previous research findings can be attributed to the minimal average differences in AQI among provinces when examined at the provincial level. In contrast to more strongly correlated explanatory variables, the influence of AQI on ABDI may have been eclipsed. Conducting an analysis of AQI at the municipal level may yield distinct outcomes.

To conclude, we have quantified the influence of nine pertinent factors on public attention, elucidated their underlying mechanisms, and established the stability of our results through goodness-of-fit tests. Consequently, there is a basis to assert that our empirical findings are robust and dependable. Nevertheless, it is important to acknowledge that there may be additional factors influencing public anxiety that were not incorporated into our model.

## 4 Discussion

China’s societal structure is experiencing significant transformations, marked by substantial alterations in societal preferences. It is essential to reduce anxiety to promote a resilient and positively oriented society (44). Gaining a deeper understanding of the mechanisms underlying anxiety and identifying the demographic groups most affected by it can considerably facilitate a smoother transition in Chinese society. The emotional state of anxiety among the public in China has been showing a consistent increase over time. The number of provinces experiencing moderate anxiety levels has been rising steadily, with even economically advanced regions like Guangdong and Jiangsu shifting into the category of severe anxiety zones. The count of inland regions falling into this category has also been increasing.

Previous research has relied heavily on survey responses to evaluate the stressors encountered by different demographic groups, which are often theoretical. However, we pursued comprehensive empirical analysis and carefully examined macro-level socioeconomic factors that impact anxiety. We collected data on nine distinct variables that may indicate a correlation with public anxiety, including GDP, the number of college graduates, and air quality metrics from all 31 provinces, ranging from 2014 to 2022. We also integrated sophisticated regression models into our analysis and accurately accounted for the spatial correlations inherent in macro-level data. We implemented OLS and GWR models, and the results show that the GWR model that utilizes spatial dimension information shows higher fitting accuracy. It is suitable for exploring the implicit connection between public anxiety and influencing factors from the spatial and temporal dimensions.

Both traditional OLS models and recently introduced GWR models have demonstrated noteworthy positive correlations among the prevalence of university graduates, internet data traffic, urbanization rate, and levels of public anxiety. Upon comparative analysis, it becomes apparent that the number of university graduates has the most significant influence on the anxiety index, highlighting their pivotal role in shaping levels of public anxiety. Our analysis indicates that university students often relocate to larger urban centers where higher education institutions are primarily located. However, owing to their relatively limited life experiences and less mature cognitive faculties compared to the general adult population, university students may face challenges in addressing their psychological well-being issues. Furthermore, our calculations demonstrate that graduates exert a more significant influence on the ABDI. This emphasizes that university graduates have become the primary demographic group experiencing public anxiety, which aligns with findings presented in other studies (45, 46).

In addition, we compared the age structure of Chinese netizens collected by CNNIC in December 2013 (47) and December 2022 (16). The results show that in December 2013, the proportions of Internet users in the age groups of 20–29, 30–39, and 40–49 were 30.7, 23.4, and 12.0%, respectively. In December 2022, the proportions of Internet users in the age groups of 20–29, 30–39, and 40–49 were 14.2, 19.6, and 16.7%, respectively. This shows that the average age of Internet users has increased significantly, which is consistent with the changing trend of public anxiety measured by Baidu search index. This may be because older netizens face greater life stress, which provides another insight into public anxiety research.

Other research has used surveys to measure public anxiety by aggregating individual anxieties, but this method does not capture all dimensions of anxiety and can be inaccurate. When it comes to measuring anxiety, using big data from the internet is an innovative approach that can overcome the limitations of existing methods. We used the most searched keyword “anxiety” to obtain the ABDI as a measure of public anxiety. This index reflects each user’s anxiety and stress, providing a more comprehensive representation of anxiety and facilitating macro-level sociological quantitative analysis. By analyzing this data across different Chinese provinces, we can investigate the macro-level mechanisms that influence public anxiety and provide valuable empirical evidence for policymakers aiming to improve public governance.

It is essential to acknowledge the limitations of this study as they can provide valuable insights for future research in this field. Baidu dominates the search engine market in China, but its coverage may vary across regions, which can impact the research outcomes. In recent years, the use of search engines such as Bing has gradually increased. People may also be more inclined to use search engines embedded in social applications such as WeChat and Weibo (17). However, since these service providers do not publish relevant index data, we cannot consider this part of search behavior. This may affect the reliability of our conclusions.

Public anxiety is influenced by various factors, including social structural changes, social risk factors, social security measures, and sociocultural values. However, this study only analyzes nine explanatory variables, including the number of university graduates, to understand public anxiety between 2014 and 2022. The study uses the BDI for the keyword “Anxiety” to evaluate the overall state of “public anxiety” at the provincial level. This approach considers the unique nature of public anxiety, which differs from individual-level anxiety. Individuals can express their anxiety through the internet while maintaining their privacy, potentially providing a more accurate reflection of anxiety levels.

However, there are concerns about sample bias, as individuals with internet access may not be representative of the broader societal landscape. Nevertheless, given Baidu’s widespread reach and the increasing ubiquity of the internet, we believe that using the BDI to measure the extent of “public anxiety” can effectively capture the overarching trends. In this context, the BDI is a valuable tool for further exploring the spatial and temporal distribution of anxiety among university students and other societal segments, as well as its influencing factors.

## 5 Conclusion

This study collected BDI data on the levels of anxiety in the public in each of China’s 31 provinces, using the keyword “Anxiety” between 2014 and 2022. In addition to this, various socioeconomic and environmental factors were included in the dataset to create a panel dataset at the provincial level. This study aimed to analyze the distributions, trends, and causes of public anxiety during China’s period of social transformation.

The analysis results show that the public anxiety levels in China have been on a consistent rise, particularly in Eastern and Central-South China. The results obtained by the GWR model that considers spatial correlation and dependence effects provide more convincing evidence than the OLS model. There are strong positive correlations between the levels of “public anxiety” at the provincial level and three key determinants: the number of university graduates, internet data traffic, and the rate of urbanization. These determinants exert varying degrees of influence, ranging from high to low.

There has been a concerning rise in instances of self-harm and suicide among graduating university students. This issue requires urgent attention from both the public and government authorities. To address this pressing concern, educational institutions and the government must prioritize increased investments in mental health education for university students, expand the pool of mental health counselors within universities, and establish targeted and robust mechanisms for monitoring and improving mental health issues.

## Data availability statement

The original contributions presented in the study are included in the article/Supplementary material, further inquiries can be directed to the corresponding authors.

## Author contributions

TX: Conceptualization, Data curation, Formal analysis, Methodology, Writing – original draft. ZH: Software, Visualization, Writing – original draft. YT: Formal analysis, Supervision, Writing – review & editing. TT: Funding acquisition, Project administration, Supervision, Writing – review & editing.
