# Supplementary material for: Analysis of the situations and influencing factors of public anxiety in China: based on Baidu index data
Source: Front Public Health. 2024 Apr 24;12:1360119. doi: 10.3389/fpubh.2024.1360119 (PMC11077890; doi:10.3389/fpubh.2024.1360119)
Supplement: Supplementary file 1 [file Data_Sheet_1.PDF]

Supplementary Material

1 Supplementary Figures and Tables······**1**

1.1 Supplementary Figures······**1**

Supplementary Figure 1. Northern China's provincial-level ABDI during 2014–2022. ······1

Supplementary Figure 2. Northeastern China's provincial-level ABDI during 2014–2022.······2

Supplementary Figure 3. Eastern China's provincial-level ABDI during 2014–2022. ······3

Supplementary Figure 4. Central-Southern China's provincial-level ABDI during 2014–2022.······4

Supplementary Figure 5. Southwestern China's provincial-level ABDI during 2014–2022.······5

Supplementary Figure 6. Northwestern China's provincial-level ABDI during 2014–2022.······6

1.2 Supplementary Tables······**7**

Supplementary Table 1. ABDI of 31 provinces in Chinese Mainland during 2014–2022.······7

Supplementary Table 2. Variables' statistical description and VIF value.······9

Supplementary Table 3. OLS and GWR model fitting results and comparison.······11

1 Supplementary Figures and Tables

1.1 Supplementary Figures

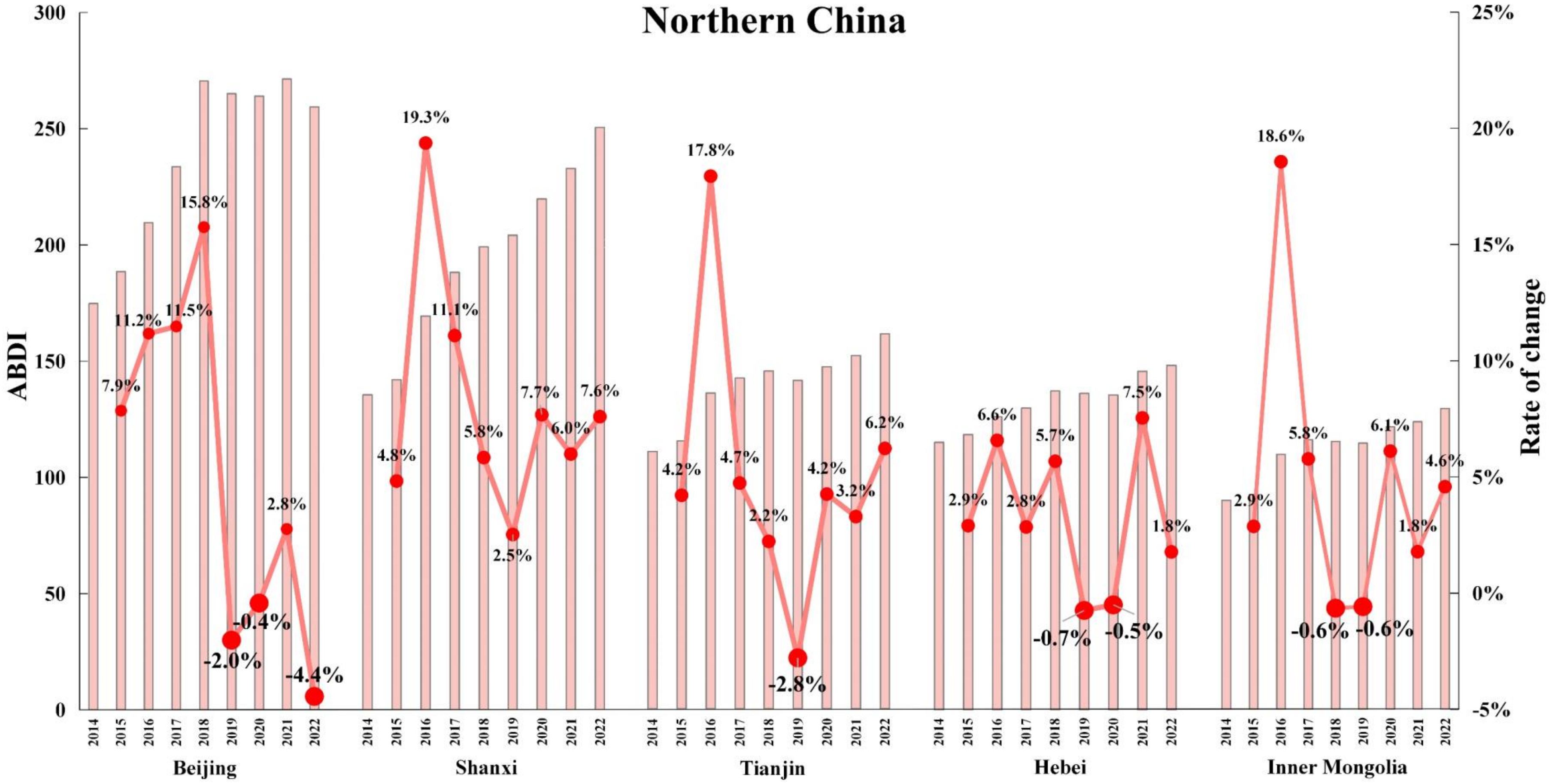

Supplementary Figure 1. Northern China's provincial-level ABDI during 2014–2022.

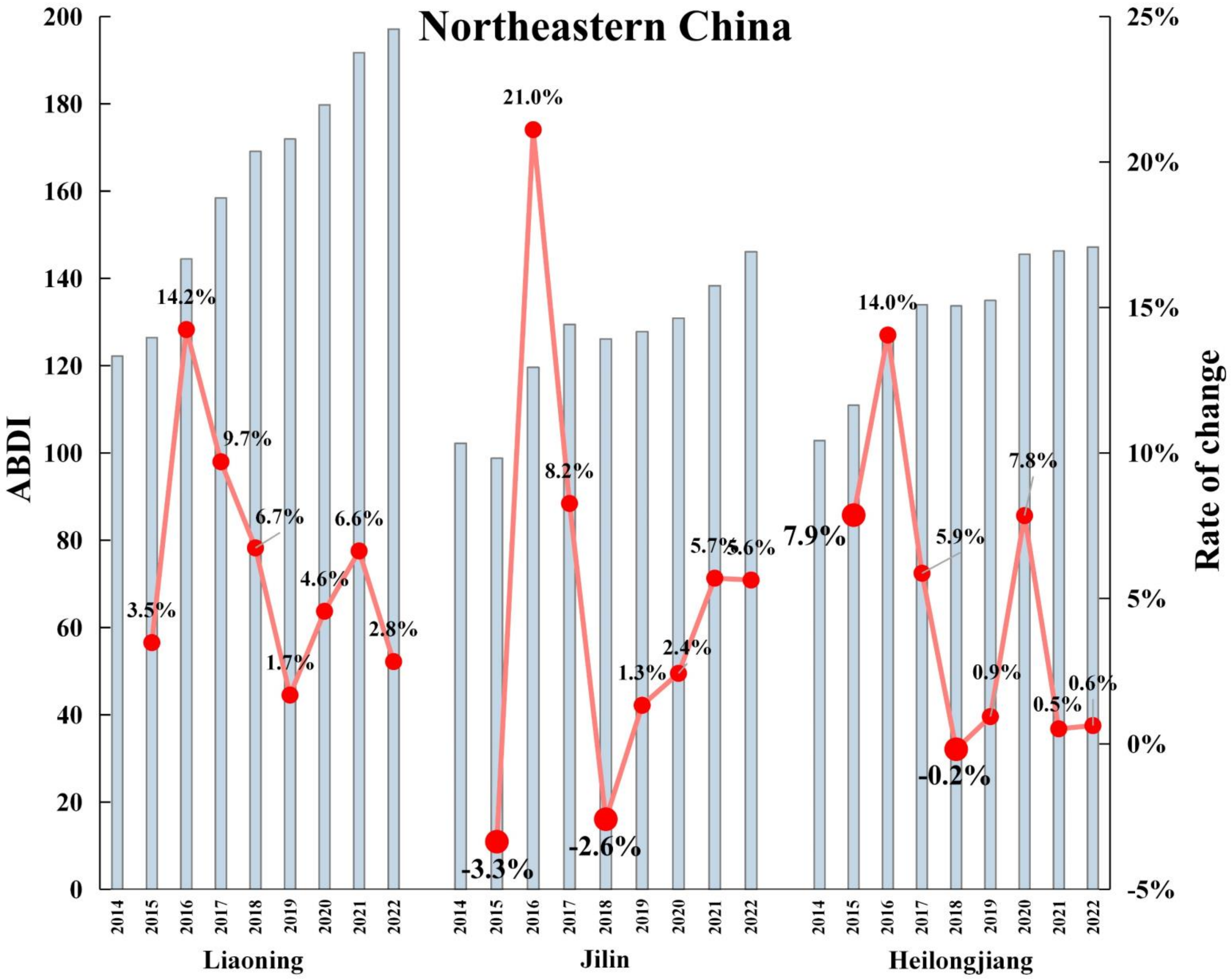

Supplementary Figure 2. Northeastern China's provincial-level ABDI during 2014–2022.

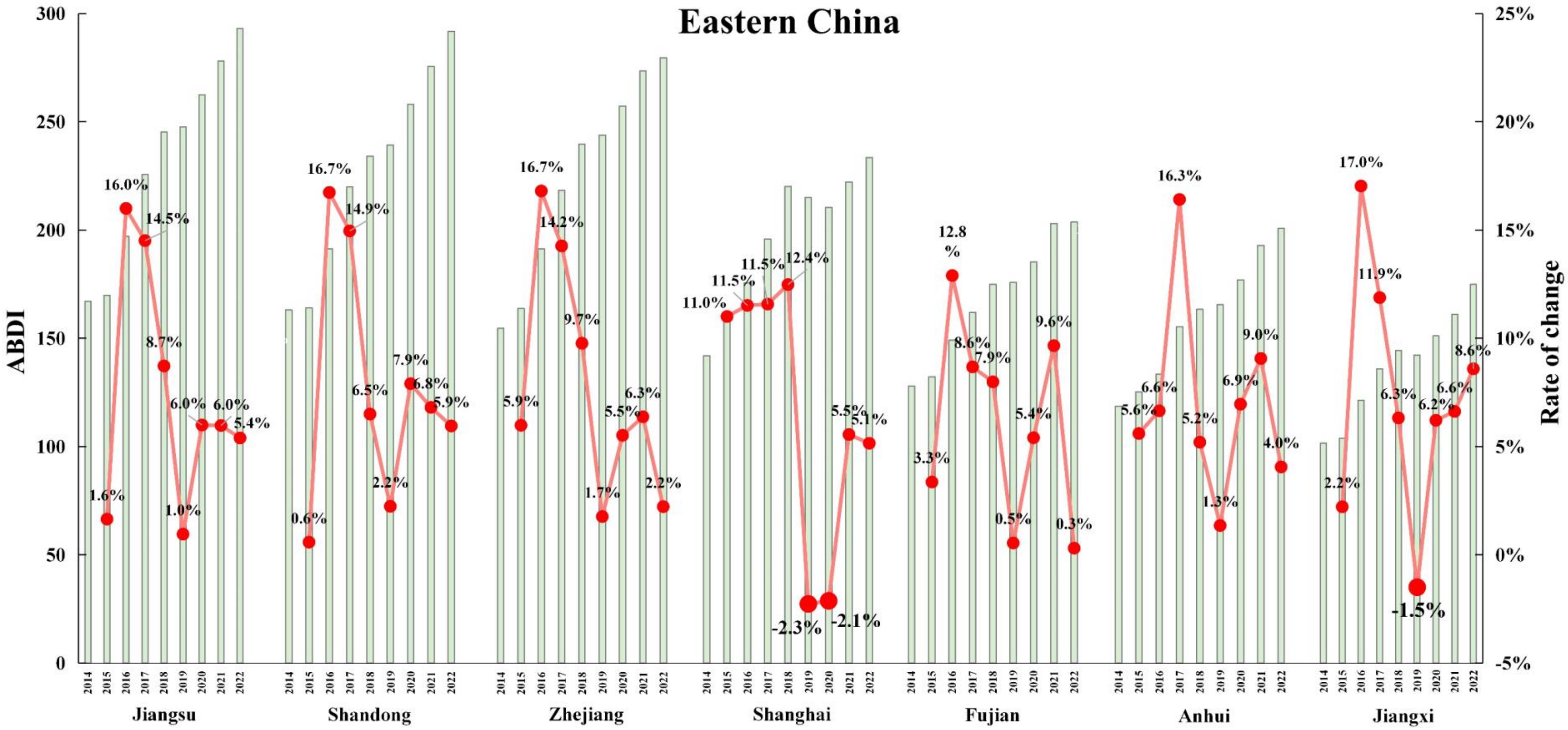

Supplementary Figure 3. Eastern China's provincial-level ABDI during 2014–2022.

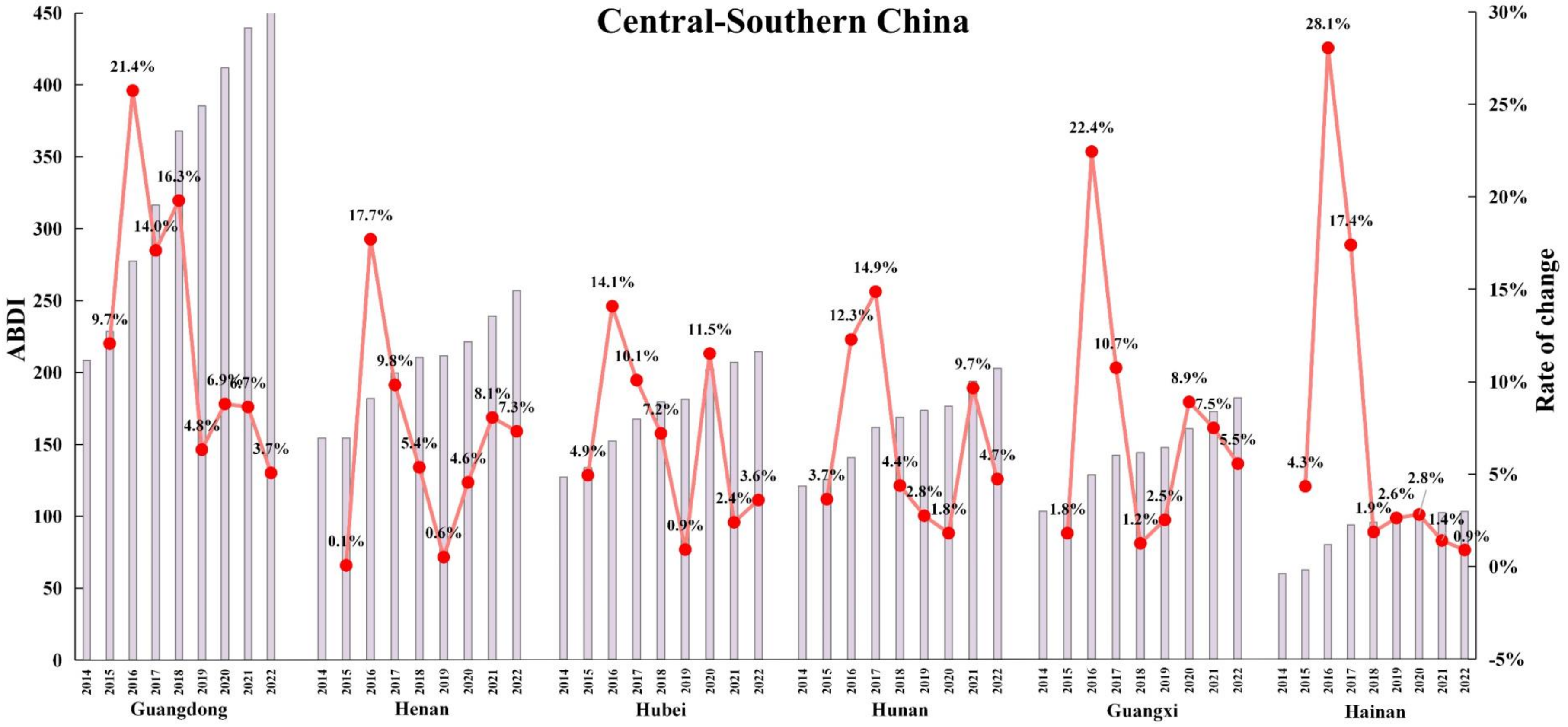

Supplementary Figure 4. Central-Southern China's provincial-level ABDI during 2014–2022.

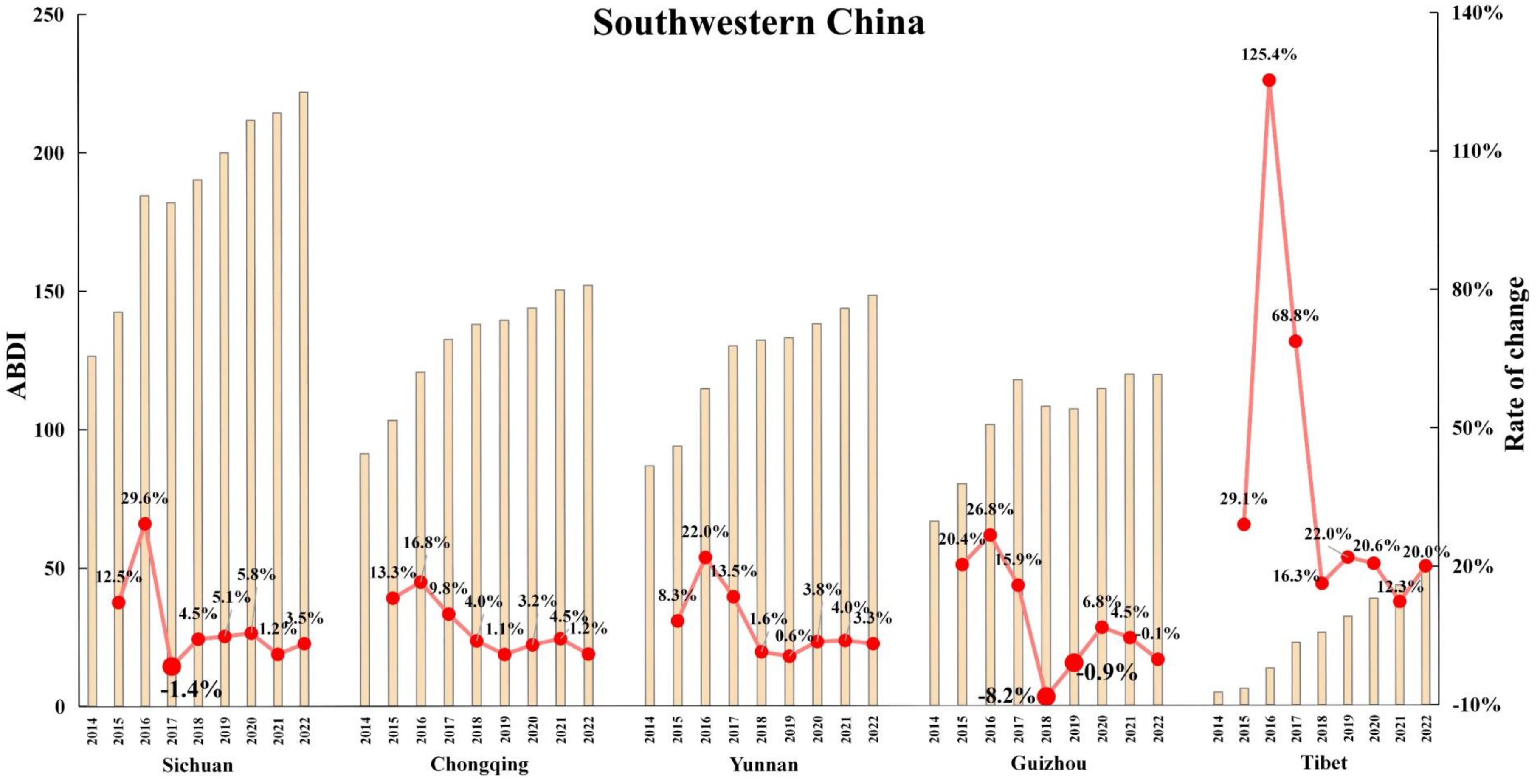

Supplementary Figure 5. Southwestern China's provincial-level ABDI during 2014–2022.

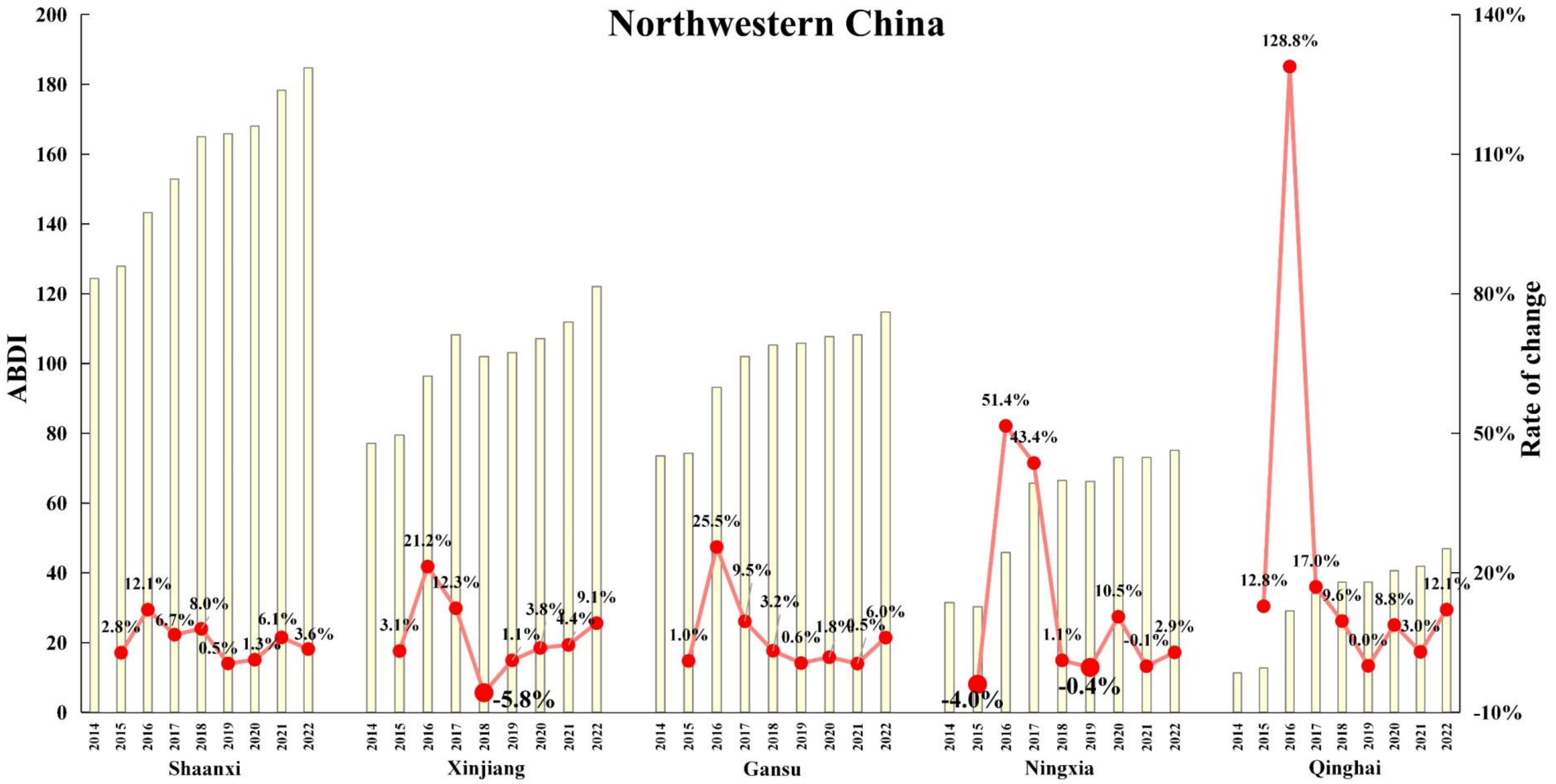

Supplementary Figure 6. Northwestern China's provincial-level ABDI during 2014–2022.

1.2 Supplementary Tables

Supplementary Table 1. ABDI of 31 provinces in Chinese Mainland during 2014–2022.

Province	2014	2015	2016	2017	2018	2019	2020	2021	2022
Beijing	174.75	188.50	209.58	233.67	270.50	265.08	264.00	269.55	271.92
Tianjin	114.75	118.08	125.83	129.42	136.75	135.75	135.08	138.36	139.42
Hebei	135.08	141.58	168.92	187.58	198.50	203.50	219.08	222.73	223.42
Shanxi	110.33	114.92	135.42	141.75	144.83	140.75	146.67	150.27	149.00
Inner Mongolia	89.92	92.50	109.67	116.00	115.25	114.58	121.58	125.00	124.50
Liaoning	122.17	126.42	144.42	158.42	169.08	171.92	179.75	184.27	184.25
Jilin	102.00	98.58	119.33	129.17	125.83	127.50	130.58	133.73	135.08
Heilongjiang	102.83	110.92	126.50	133.92	133.67	134.92	145.50	149.27	149.00
Shanghai	141.50	157.00	175.00	195.17	219.42	214.42	209.83	214.73	214.83
Jiangsu	167.08	169.83	197.00	225.58	245.25	247.58	262.42	266.73	266.33
Zhejiang	154.17	163.33	190.67	217.75	238.92	243.08	256.42	262.91	261.92
Anhui	118.25	124.83	133.08	154.83	162.83	165.00	176.42	180.82	182.25
Fujian	127.50	131.75	148.67	161.50	174.33	175.25	184.67	189.91	190.08
Jiangxi	101.50	103.75	121.42	135.83	144.42	142.25	151.08	155.36	153.25
Shandong	162.58	163.50	190.75	219.17	233.33	238.50	257.25	262.36	261.42
Henan	153.83	154.00	181.25	199.08	209.83	211.00	220.67	226.55	225.00
Hubei	126.58	132.83	151.50	166.75	178.75	180.42	201.17	206.55	201.25
Hunan	120.50	124.92	140.25	161.08	168.17	172.83	176.00	180.64	181.17
Guangdong	208.42	228.58	277.50	316.33	367.92	385.42	411.92	422.09	419.83
Guangxi	102.50	104.33	127.67	141.33	143.08	146.67	159.67	163.73	162.83
Hainan	59.50	62.08	79.50	93.33	95.08	97.58	100.33	102.55	102.83
Chongqing	90.83	102.92	120.17	132.00	137.33	138.83	143.25	147.55	148.92
Sichuan	126.50	142.33	184.42	181.92	190.17	199.92	211.58	216.64	213.17
Guizhou	66.25	79.75	101.08	117.17	107.58	106.67	113.92	115.73	116.58

Yunnan	86.33	93.50	114.08	129.50	131.58	132.42	137.42	142.18	141.83
Tibet	4.58	5.92	13.33	22.50	26.17	31.92	38.50	39.64	39.58
Shaanxi	124.33	127.83	143.25	152.83	165.00	165.83	168.00	171.18	172.67
Gansu	73.25	74.00	92.83	101.67	104.92	105.50	107.42	110.18	109.25
Qinghai	16.92	19.08	43.67	51.08	56.00	56.00	60.92	61.09	60.25
Ningxia	31.42	30.17	45.67	65.50	66.25	66.00	72.92	74.27	72.75
Xinjiang	76.83	79.25	96.08	107.92	101.67	102.83	106.75	109.18	109.50

Supplementary Table 2. Variables' statistical description and VIF value.

Year		Variables								
		GDPP	PD	UR	NCG	IDT	PT	AT	UR	AQI
2014	**Mean**	46895.81	457.44	3.28	21.27	6651.10	913.65	14.41	55.91	95.44
	**Std. Deviation**	20729.00	713.10	0.65	13.65	5650.01	568.11	5.10	13.46	25.03
	**Minimum**	25101.00	2.72	1.30	0.91	527.94	169.20	5.10	26.15	41.08
	**Maximum**	106732.00	3891.78	4.50	47.87	29287.79	2234.00	24.70	89.30	160.83
	**VIF**	8.34	3.06	1.45	6.22	4.00	6.20	3.92	5.83	4.85
2015	**Mean**	49498.29	459.07	3.26	21.96	13507.91	1011.63	14.56	57.40	89.44
	**Std. Deviation**	22221.01	711.69	0.67	13.98	11011.42	660.36	5.05	12.86	22.44
	**Minimum**	25946.00	2.76	1.40	0.95	738.21	191.00	5.60	28.79	42.83
	**Maximum**	113692.00	3877.58	4.50	48.41	55458.73	2471.90	25.30	88.53	135.08
	**VIF**	7.26	3.15	1.58	8.86	5.03	6.24	4.10	5.43	6.74
2016	**Mean**	53260.00	461.76	3.26	22.71	30250.36	1128.92	14.60	58.89	88.08
	**Std. Deviation**	24391.89	714.48	0.67	14.46	23889.14	663.18	5.17	12.34	21.43
	**Minimum**	27396.00	2.85	1.40	0.92	1149.19	264.90	5.00	31.47	43.08
	**Maximum**	123391.00	3891.78	4.20	50.91	126199.37	2939.70	24.60	89.01	135.67
	**VIF**	7.04	3.16	1.54	6.12	4.02	9.02	3.52	4.98	5.55
2017	**Mean**	58706.74	462.63	3.18	23.74	79334.84	936.49	14.73	60.27	88.00
	**Std. Deviation**	26815.02	713.41	0.64	15.10	61513.60	532.94	5.11	11.88	21.04
	**Minimum**	29103.00	2.92	1.40	0.90	2420.17	211.30	5.10	33.24	44.25
	**Maximum**	136172.00	3890.20	4.20	57.12	328666.06	2067.40	24.60	89.09	133.92
	**VIF**	6.85	2.99	1.49	4.11	3.45	7.66	5.33	5.17	3.37
2018	**Mean**	64366.13	463.64	3.11	24.30	228710.94	950.84	14.51	61.48	72.98
	**Std. Deviation**	29400.73	715.09	0.57	15.67	167548.08	510.15	5.19	11.57	16.89
	**Minimum**	32178.00	2.96	1.40	0.93	10618.37	280.20	5.10	33.90	39.42

Year		Variables								
		GDPP	PD	UR	NCG	IDT	PT	AT	UR	AQI
	Maximum	150962.00	3904.40	4.00	58.59	845841.16	2135.30	24.40	89.13	112.17
	VIF	6.12	2.94	1.70	6.61	4.28	5.67	3.93	4.42	3.98
2019	Mean	68867.10	465.19	2.95	24.47	393522.60	889.14	14.73	62.63	79.23
	Std. Deviation	31067.75	716.77	0.59	15.83	274109.94	540.77	5.12	11.33	18.19
	Minimum	34707.00	3.02	1.30	0.99	34381.28	145.50	5.90	34.63	46.67
	Maximum	161776.00	3913.87	4.20	59.34	1379370.96	2459.30	25.80	89.24	114.00
	VIF	7.64	3.20	1.61	6.77	4.72	6.34	3.22	6.04	5.08
2020	Mean	70658.23	466.34	3.29	25.72	534090.88	976.09	14.65	63.72	73.20
	Std. Deviation	31340.34	718.83	0.58	16.72	367614.49	545.56	5.21	11.05	16.31
	Minimum	35848.00	3.07	2.10	0.98	56444.15	186.50	5.40	35.79	42.58
	Maximum	164158.00	3924.91	4.60	63.82	1840549.20	2140.70	25.30	89.31	103.00
	VIF	5.24	2.75	1.24	7.13	4.43	3.37	2.57	4.97	4.31
2021	Mean	79619.00	466.44	3.07	26.66	714942.72	1017.06	14.98	64.51	72.27
	Std. Deviation	34923.92	718.84	0.65	17.43	502506.81	495.19	5.23	10.80	16.14
	Minimum	41046.00	3.07	1.80	0.91	77993.90	146.40	5.50	36.61	42.50
	Maximum	183980.00	3926.49	4.50	67.84	2466758.80	1936.80	25.10	89.31	101.08
	VIF	4.75	2.60	1.21	6.68	4.87	4.11	3.43	4.20	2.63
2022	Mean	84461.26	457.44	2.99	31.20	844382.81	477.42	15.15	65.00	53.80
	Std. Deviation	36090.00	713.10	0.70	19.96	604587.78	324.84	5.19	10.65	15.85
	Minimum	44968.00	2.72	1.70	1.02	93875.35	65.90	5.50	37.36	17.50
	Maximum	190313.00	3891.78	4.50	77.80	2956007.26	1537.40	24.50	89.33	80.58
	VIF	4.53	2.65	1.28	4.75	4.91	1.78	2.12	4.10	1.88

Supplementary Table 3. OLS and GWR model fitting results and comparison.

Year	Model	Parameter	Variables									Model Evaluation Index		
			Ln GDPP	Ln PD	Ln UER	Ln NCG	Ln IDT	Ln PT	Ln AT	Ln UR	Ln AQI	R^2	Adjusted R^2	AICc
2014	OLS	Coefficient	–0.4639	0.0184	–0.1519	0.5233***	0.1819	–0.1686	0.1049	1.5274***	–0.3555	0.9561	0.9373	7.8929
		t–statistic	–1.1167	0.2937	–0.9691	4.4191	1.5422	–1.662	0.5440	3.9930	–1.4777			
	GWR	Coefficient	–0.1548	0.0740	–0.2492	0.5888***	0.0615	–0.1395	0.0731	0.8957*	–0.3979	0.9879	0.9779	–15.2757
		t–statistic	–0.5877	1.0625	–1.2296	4.6093	0.4685	–1.3189	0.3431	1.9126	–1.5577			
2015	OLS	Coefficient	–0.2918	0.0080	–0.1214	0.5146***	0.1341	–0.0915	0.1286	1.4414***	–0.2189	0.9503	0.9290	9.3794
		t–statistic	–1.302	0.1257	–0.7095	4.5187	1.1749	–0.7611	0.5698	3.3807	–0.813			
	GWR	Coefficient	–0.0678	0.1185	–0.2074	0.5571***	0.0680	–0.118	–0.0501	0.8022**	–0.4209	0.9797	0.9628	–1.7546
		t–statistic	–0.2617	1.6928	–1.127	4.7595	0.5731	–0.9543	–0.2024	2.6074	–1.5201			
2016	OLS	Coefficient	–0.1465	–0.0199	–0.1707	0.3245***	0.2221***	–0.1196	0.1699	1.0923***	–0.1389	0.9546	0.9351	–8.1983
		t–statistic	–0.8668	–0.4223	–1.3999	3.8402	2.7897	–1.0875	1.2470	3.0912	–0.6811			
	GWR	Coefficient	–0.1143	0.0026	–0.1588	0.3289***	0.2116***	–0.1047	0.1058	0.9641***	–0.1467	0.9671	0.9465	–12.7508
		t–statistic	–0.7011	0.0574	–1.3451	4.0494	2.7600	–0.9896	0.7909	2.8123	–0.7481			
2017	OLS	Coefficient	0.0345	0.0158	–0.2187**	0.2136***	0.2117***	0.0392	0.0043	0.5923**	0.1357	0.9724	0.9606	–31.8391
		t–statistic	0.3467	0.4864	–2.5027	3.8425	4.1710	0.5658	0.0429	2.4249	0.9977			
	GWR	Coefficient	0.0398	0.0277	–0.1989**	0.2015***	0.2186***	0.0421	–0.0032	0.5475**	0.1334	0.9801	0.9682	–38.1146
		t–statistic	0.4194	0.8875	–2.3733	3.7679	4.5056	0.6392	–0.0337	2.3441	1.0308			
2018	OLS	Coefficient	0.1039	0.0457	–0.2984***	0.164**	0.2407***	0.0101	–0.0646	0.5747**	0.0765	0.9762	0.9660	–35.2661
		t–statistic	1.0866	1.4576	–3.2956	2.6688	4.1061	0.1561	–0.7088	2.5573	0.5215			
	GWR	Coefficient	0.1034	0.0462	–0.2914***	0.2396***	0.2435***	0.0092	–0.063	0.5754**	0.0774	0.9870	0.9745	–44.7301
		t–statistic	1.0808	1.4705	–3.2121	3.7917	4.1508	0.1416	–0.6899	2.5588	0.5281			

Year	Model	Parameter	Variables									Model Evaluation Index		
			Ln GDPP	Ln PD	Ln UER	Ln NCG	Ln IDT	Ln PT	Ln AT	Ln UR	Ln AQI	R^2	Adjusted R^2	AICc
2019	OLS	Coefficient	0.1488	0.0639	–0.2117**	0.1517*	0.2609***	0.0562	–0.1101	0.4274	0.1068	0.9629	0.9470	–23.5351
		t–statistic	1.4298	1.6287	–2.0705	1.7570	3.1459	0.9457	–0.9331	1.5854	0.6496			
	GWR	Coefficient	0.1478	0.0627	–0.2052*	0.2065**	0.2652***	0.0554	–0.1079	0.4322*	0.1095	0.9743	0.9549	–30.2415
		t–statistic	1.4193	1.5960	–2.0052	2.0552	3.1964	0.9312	–0.9137	1.7019	0.6654			
2020	OLS	Coefficient	0.1930	0.0473	–0.2915*	0.2045*	0.2204**	–0.0018	–0.0415	0.4763	0.0082	0.9540	0.9343	–19.1137
		t–statistic	1.6816	1.1397	–1.9869	1.8768	2.1179	–0.0258	–0.359	1.6559	0.0439			
	GWR	Coefficient	0.0558	0.0233	–0.23	0.2492**	0.2785**	–0.044	0.0738	0.9178**	0.0144	0.9769	0.9575	–32.6576
		t–statistic	0.3905	0.4111	–1.2679	2.4017	2.4297	–0.5559	0.5307	2.3968	0.0704			
2021	OLS	Coefficient	0.1770	0.0609	–0.0832	0.1223	0.3122***	0.0166	–0.113	0.4508	–0.0234	0.9477	0.9253	–15.0183
		t–statistic	1.4059	1.3602	–0.6367	1.2221	2.9214	0.2314	–0.917	1.3713	–0.1501			
	GWR	Coefficient	0.1756	0.0560	–0.114	0.1917**	0.3209***	0.0111	–0.0899	0.4877*	–0.0147	0.9549	0.9287	–18.6249
		t–statistic	1.4172	1.2706	–0.8771	2.0453	3.0508	0.1567	–0.739	1.7053	–0.0963			
2022	OLS	Coefficient	0.2375	0.1085	–0.2877	0.1250	0.2983***	0.0133	–0.2249	0.4452	–0.0559	0.9523	0.9319	-19.7487
		t–statistic	1.6661	1.4084	–1.4213	1.4981	3.3017	0.3286	–1.2283	1.5377	–0.6322			
	GWR	Coefficient	0.2353	0.1043	–0.2728	0.2211**	0.3007***	0.0123	–0.2227	0.4584*	–0.0529	0.9752	0.9539	-33.3679
		t–statistic	1.6741	1.6061	–1.4747	2.1949	3.3870	0.3025	–1.2245	1.8003	–0.5596			

Notes: ***, ** and * represent the 1%, 5% and 10% significance levels, respectively.
